# Supplementary material for: Harnessing synthetic lethality to predict the response to cancer treatment
Source: Nat Commun. 2018 Jun 29;9:2546. doi: 10.1038/s41467-018-04647-1 (PMC6026173; doi:10.1038/s41467-018-04647-1)
Supplement: Supplementary file 1 — Supplementary Information [file 41467_2018_4647_MOESM1_ESM.pdf]

Supplementary Information

Harnessing synthetic lethality to predict the response to cancer treatment

Joo Sang Lee, Avinash Das, Livnat Jerby-Arnon, Rand Arafeh, Noam Auslander, Matthew Davidson, Lynn McGarry, Daniel James, Arnaud Amzallag, Seung Gu Park, Kuoyuan Cheng, Welles Robinson, Dikla Atias, Chani Stossel, Ella Buzhor, Gidi Stein, Joshua J. Waterfall, Paul S. Meltzer, Talia Golan, Sridhar Hannenhalli, Eyal Gottlieb, Cyril H. Benes, Yardena Samuels, Emma Shanks, Eytan Ruppin

Supplementary Note 1 .... **1**
Evaluating clinical relevance of experimentally identified SL interactions 1
Identifying under-represented candidate SL pairs 3
Identifying phylogenetically linked candidate SL pairs 3
ISLE-identified cSL pairs predict patient survival 4
Validating ISLE with *in vitro* drug response data 4
Validating ISLE with *in vivo* drug response data 4
Benchmarking ISLE with DREAM7 challenge 5
Experimentally testing ISLE-based gene essentiality and drug prioritization prediction 6
Experimentally testing ISLE-based drug synergy prediction in patient-derived cell lines 7
Predicting *erlotinib* response in lung cancer patients 8
Threshold for gene underactivation 8
Implementation of DAISY 9
Supplementary Note 2 .... **10**
Supplementary Figures **11**
Supplementary References **19**

Supplementary Note 1

Evaluating clinical relevance of experimentally identified SL interactions

To investigate the clinical relevance of the known SL pairs (Initial Set I), we first collected the **experimentally identified SLi from 17** *in vitro* focused SL screens[1-17], surveying altogether **154,707** pairwise interactions, that resulted in **6,033** true positive SL pairs (**Supplementary Data 1**). We excluded from the screens those interactions that focus on activating driver mutation or copy number amplification, such as KRAS[6,9], PTTG1[16], and MYC[8], resulting in 4,457 candidate pairs tested in 8 different cancer types with 4,252 unique pairs. We then analyzed them using step I and II of ISLE (Methods) to determine the clinical relevance of them. Specifically, we checked (Step I) whether the co-inactivation of a candidate SL (determined by gene copy number and transcriptomic data) is underrepresented among the tumors. This criterion arises from the premise that SLi pairs, being deleterious to the tumor, are likely to be under negative selection. (Step II) We then further evaluated whether the co-inactivation of a given candidate SL is associated with better prognosis in patients – we expect

that a true SL when both its partnering genes are down-regulated, will have a detrimental effect on tumor growth and thus improve overall patient survival.

For example, consider the case of evaluating the clinical relevance of the SLi between AKT1 and BRCA1, which is frequently mutated in breast cancer. Experimentally, this can be interrogated in breast cancer cell lines via (A) AKT1 shRNA knockout in BRCA1-isogenic cancer cell line, or (B) double AKT1-BRCA1 knockout screen. In our evaluation of step I and step II, the *co-inactivation* state was determined in two different ways following the respective experimental screens (A and B). (A) In the case of an isogenic cell line, the co-inactivation of the gene pair is determined based on (1) BRCA1 mutation and AKT1 down-regulation by using AKT1 expression, and (2) BRCA1 mutation and AKT1 copy number loss using its SCNA values. (B) Similarly, in the case of a double knockout screen, the co-inactivation is determined based on (3) down-regulation of both genes using their expression, and (4) copy number loss of both genes using their SCNA values. Then, we evaluate clinical relevance in (i) pancancer samples and in (ii) breast cancer samples of TCGA cohort, resulting in 4 different statistical tests for a given experimentally identified SL pair. That means, a candidate SL pair that is identified by an isogenic cell line screen (or double knockout screen) are evaluated by steps I and II using both pancancer and breast cancer data, where the co-inactive state is determined by 1 and 2 (or 3 and 4, respectively). If significance was observed in at least one of these four tests, the *in vitro* SL pair was marked as significant for each of steps I and II, independently. We (i) included mutation data and (ii) considered pancancer and cancer type specific significance not to miss any important clinically relevant SL pairs. The total number of pairs that pass each of step I and II of ISLE are summarized in **Supplementary Data 5**.

However, SL pairs identified from experimental screens have the following limitations. The SL partners of a gene that are identified by conducting dropout screen in two different cell lines of the same cancer type differ significantly from each other. For example, SL-partners of KRAS in DLD1[12] and HCT116[20] colorectal cancer cell lines show a small (5%) overlap. Specifically, out of 14,657 candidates SL-partners tested in both screens, 502 and 448 SL-partners show significance in each cell line, while only 27 shows signal in common in both cell lines. The second limitation of isogenic or double knockout screens are low coverage, i.e, only few SL partners of few genes are determined experimentally.

To overcome above limitations, we inferred candidate SL pairs by capitalizing on the large-scale single gene knockout screens. We inferred the candidate SL pairs (Initial Set II) from 5 large-scale shRNA/sgRNA single gene knockout datasets (see Methods), and we select a gene pair significant if it passes false discovery correction in at least one of the 5 datasets (FDR<0.2). This is because each of the 5 shRNA/sgRNA datasets covers a different range of cancer types and genes. For example, the latest Achilles shRNA screening[18] covers 216 cell lines of 19 cancer types but the data includes only 4,898 gene knock-outs (because only those genes passed the quality control), whereas the previous version of Achilles[19] covers less number of cell lines and cancer types, but covers more gene knock-downs (N=6,885). Similarly, a recent shRNA screening from Marcotte et al.[20] covers

essentiality scores of all 20,000 protein-coding genes but only for breast cancer cell lines, while their old screening[21] includes the essentiality score of 15,110 genes but covers breast, pancreatic, and ovarian cancer cell lines. The recent sgRNA screen includes all protein-coding genes in 33 cell lines[22]. Hence we included a candidate gene-pair if it is statistically significant in at least one dataset. For the inferred SL pairs that do not belong to Initial Set I, we applied standard ISLE steps I and II to evaluate the clinical relevance (as described in the Methods, **Supplementary Data 5**).

Identifying under-represented candidate SL pairs

To determine a gene to be underactive, we controlled for the different basal expression levels in each cancer type. Specifically, we determine inactive genes in each cancer type separately, which controls for cancer-type-specific different basal expression levels, and performed a single hypergeometric test on all cancer types together, as follows. First, we compared the expression level of that gene to its expression in samples of the same cancer type, and denoted the gene is down-regulated if it falls below 1/3-quantile. Second, once the down-regulated gene is identified in each cancer type, we performed a hypergeometric test for each pair of genes across all samples, combining all cancer types, to assess whether a given pair is observed jointly down-regulated significantly less than expected. We applied the same approach for mRNA expression level and SCNA data and used FDR<0.2 based on Benjamini-Hochberg[23] for both cases.

Identifying phylogenetically linked candidate SL pairs

The purpose of including the gene phylogeny screen is to further refine SL prediction. This enables us to better distinguish the true SL-partners from spurious ones because many gene co-expression and correlated SCNA of proximal genes.

To determine the phylogenetic distance between a pair of genes, we used non-negative matrix factorization (NMF) to consider the structure of the tree of life. Let's consider two cases of phylogenetic profiles of genes A and B, each having five orthologues in different species: (Case 1) the five orthologues of genes A and B are widely spread in multiple domains of life encompassing archaea, bacteria and eukaryotes, and (Case 2) the five orthologues of genes A and B are focused on primates. Simple similarity measures such as Hamming distance will find that the two cases have the same phylogenetic distance. However, they are vastly different when considering the structure of the tree of life, with Case 1 obviously being much more conserved. To capture this important difference we used NMF, following a standard procedure in the literature[24].

To determine the threshold for phylogenetic similarity, we used the experimentally identified gold standard SL set (Initial Set I as described in Methods) - a total of 154,707 pairs, among which 6,033 pairs constitute the positive set (and the rest belongs to the negative set). We first identified the phylogenetic similarity of all the pairs and found the cut-off value of phylogenetic distance that best separates the positive set and negative set, with the objective being minimizing the p-value in the hypergeometric test. The optimal value was the top 50-percentile of the randomly selected gene pairs (a phylogenetic distance of 10.5). ISLE uses this criterion to determine phylogenetically linked pairs.

ISLE-identified cSL pairs predict patient survival

We investigated the performance of the ISLE-identified cSL pairs in predicting patient survival in an independent breast cancer dataset (Molecular Taxonomy of Breast Cancer International Consortium; METABRIC)[25]. To this end, we characterized a cSL pair to be *functionally active* if both its partnering cSL genes are underactive in that sample (determined from that sample's transcriptomic profile). Analyzing the METABRIC collection, we found that tumors with many functionally active cSLi exhibit significantly better patient survival than tumors with few functionally active cSLi, as predicted (Cox hazard ratio= 1.35 ($P<3.45E-10$)) controlling for age, metastasis to lymph nodes and breast cancer. **Supplementary Figure 10B** demonstrates that the signal is robust versus different thresholds for dividing patient groups in KM analysis, where the patients with high cSL-score consistently show better prognosis than the patients with low cSL-score (logrank $P<1E-6$ in all thresholds), demonstrating ISLE-based prediction does not strongly depend on the parameter choices. This analysis was performed using the core cSL network by applying FDR<0.1 to ISLE pipeline (**Figure 1B**).

Validating ISLE with *in vitro* drug response data

In this analysis, we focused on the five *inhibitor* compounds whose response is highly variable across different cell lines (standard deviation within top 75-percentile). We used the cSL-pair-score of the P - T pair to predict the association between the low expression of P and better response to the drug targeting T (i.e. the labels, 1 if the low expression of P is associated with the better response to the drug targeting T, 0 otherwise) using Wilcoxon rank sum test with FDR<0.2 and fold change >0.35, which leads to 2.3% of P - T pairs to be the positive set (1527 out of a total of 66,664 pairs), and all the remaining P - T pairs in the negative set.

Validating ISLE with *in vivo* drug response data

For *in vivo* analysis, we analyzed the large-scale mouse xenograft dataset, which collects 36 single drug response screening of 375 mouse samples in 15 cancer types[26]. Following Procedure 1 (see 'ISLE-based drug response prediction' in Methods) using drug-cSL network, we evaluated our prediction (cSL-score deduced from their transcriptomic and copy number profiles in the pre-treatment tumor samples) vs. experimentally measured drug response. Specifically, we marked a gene is inactive when both its expression and copy number is less than 1/3 quantile across samples in each cancer type. Based on the *in vivo* pathological drug response annotation, the samples were divided into responders (CR, PR, SD) vs. non-responders (PD) and their cSL-score were compared using Wilcoxon rank sum test. We focused on 7 drugs that have a limited number of drug targets (≤3), sufficient variability in best average response (BestAvgResponse)[26] across different samples per drug (variance >10-percentile), and sufficient sample size (at least 4 responders and 10 nonresponders). We further filtered out the drugs whose average response rate and maximal response rate have large values (more than 50% and 275%, respectively) in terms of the same response measure (BestAvgResponse), and focused on the drugs that were primarily used in a single cancer type. In addition, we also tested our prediction against *in vivo* Progression Free Survival (PFS) data, which measures the time for the tumor to grow double of the baseline tumor size (see Methods in Gao *et al.*[26]). We tested whether high cSL-score is associated with improved survival using Cox regression analysis while controlling for other confounders such as cancer subtypes.

Benchmarking ISLE with DREAM7 challenge

We evaluated the prediction accuracy of ISLE in comparison to existing supervised methods tested in DREAM7 challenge for single[27] and double[28] drug response screens. For single drug response, the challenge was to predict the ranking of effectiveness of the 28 drugs in 18 test cell lines based on the drug response data provided for 28 drugs in 35 training cell lines and various types of omics data available for each training cell line. We focused our analysis on a set of 15 inhibitor compounds that have highly specific targets (N_{target}<3) and significant SL partners predicted by ISLE. We followed Procedure 1 (see 'ISLE-based drug response prediction' in Methods) using cSL network to make ISLE-based drug response prediction with *cSL-score*, which denotes the number of its inactive cSL-partners, as inferred from the cell-line's transcriptomic data. We resort to both gene expression and RNAseq data to increase the coverage of cell lines because a particular data type was not available across all cell lines. We then evaluated and compared the performance using weighted probabilistic concordance index (wpc-index), that is the collective measure of concordance-index for each drug, taking the variance of the response to individual drugs into account, as defined in Costello *et al.*[27]. The resulting ISLE-based prediction ranks fairly high versus the top supervised algorithms reported in DREAM7[27] (**Figure 2C (**left columns)).

For drug combination, the task was to predict drug synergism in a single cancer cell line using transcriptomic profiles before and after the treatment. We hypothesized that a pair of drugs will be synergistic if there exists a

strongly predicted SL between any of their gene targets (Methods). To this end, we used the ISLE-inferred SL significance between the drug targets as the predictor for synergism (cSL-pair-score), focusing on 12 inhibitor compounds (66 combinations) excluding 2 non-specific drugs from the data. We followed Procedure 2 (see 'ISLE-based drug response prediction' in Methods) to make synergistic compound prediction. For multi-target drugs, we assigned the minimum cSL-pair-score between the target genes as the synergy prediction for a given drug pair. We then evaluated and compared the performance using probabilistic concordance index (pc-index), taking the variance of the response to individual drugs into account, as defined in Bansal *et al*[28]. ISLE prediction accuracy ranks at the top compared to the top 5 algorithms reported in the DREAM7[28] (**Figure 2C** (right columns)). In both the cases, the performance of randomly selected, DAISY-identified and most importantly, ncSL partners is markedly inferior to that of ISLE (**Supplementary Figure 3**). Notably, ISLE does not require any post-treatment transcriptomic profiles that were used to train all the other competing approaches in the original challenge, making it broadly applicable for drug synergy prediction without the need of further training.

Experimentally testing ISLE-based gene essentiality and drug prioritization prediction

We show that SL-network can **predict context-specific gene essentiality** using an shRNA knockdown (KD) screening focused on known cancer drivers[29,30] in a liv7k oral cancer cell line under hypoxic and normoxic conditions. We performed an SL-based computational analysis that predicts the effects of each gene KD on cellular proliferation in each condition - this was done using cSL-score, i.e, the count of predicted cSL partners of each gene whose expression is down-regulated in that specific cell-line. Starting from the initial SL pool, an FDR threshold of 0.2 was applied to identify cSL interactions between the genes whose essentiality was measured and the candidate SL partners that are inactive in at least one replicate (since the remainder does not change cSL-score). The result presented for predicting general growth reduction are for genes that show a robust essentiality score within each condition (< variance 45-percentile in both conditions). We focused on the genes which have at least 2 SL partners to guarantee sufficient variation in cSL-score. We compared the performance of ISLE in predicting gene essentiality (**Figure 3A,** the individual t-test p-values between each bin are P=0.02, 0.06, 0.01, and 0.07, respectively) to that of DAISY, ncSL and random networks using the SL-score based on these respective networks as prediction for essentiality. DAISY and ncSL partners do not show a significant difference in essentiality between the genes of different SL-score, and the random networks (randomly assigned SL-partners) do not achieve the significance of ISLE (empirical P<1E-3). In predicting context–specific essentiality, we considered 38 experimentally differentially *essential* genes (Wilcoxon rank sum P<0.2, |log(fold change)|>0.1) that are not differentially *expressed* (|log (fold change)|<0.05) to rule out the genes that are differentially essential merely due to the differential expression. We focused on the genes whose cSL-scores are differential between the

two conditions (Wilcoxon rank sum $P<0.1$). We made an analogous comparison of ISLE's performance (**Figure 3B**) to that of DAISY, ncSL, and random networks. The fold change of cSL-scores quantified based on DAISY and ncSL networks do not show a significant correlation to the fold change of essentiality in the two different conditions, and ISLE's performance is not reached by random SL partners (empirical P<1E-3).

For the drug prioritization prediction in oral and breast cancer cell lines, the drug response was predicted as cSL-scores of drug's target genes. For conditional effectiveness of each drug, we focused on the drugs whose cSL-score is different in the two conditions (N=20). We evaluated the accuracy of the prediction in a manner analogous to that of conditional essentiality. The fold change (FC) values of predicted cSL-score and the measured drug response in hypoxia to that of normoxia show a significant correlation (Spearman R=0.36 ($P<0.01$)). The drugs whose cSL-score is higher in one condition indeed are more effective based on the measured drug response in the condition - 19 out of 20 drugs shows concordance – the same sign of log(FC) of predicted cSL-score and measured growth inhibition.

Experimentally testing ISLE-based drug synergy prediction in patient-derived cell lines

We show ISLE predicts personalized novel synergistic drug combinations. We tested the top hits of ISLE in melanoma using *patient-derived* cell lines (PDC). Starting from the drug cSL-network, we first filtered the actionable targets relevant to melanoma, prioritizing the pathway alterations associated with the key melanoma drivers[31]. We ranked the pairs using cSL-pair-score (defined in Methods) based on pancancer and melanoma ISLE screens to take into account the melanoma-specific features. We used their combined score as the final criteria to select the pairs, where averaged rank of pancancer cSL-pair-score and melanoma cSL-pair-score was normalized between 0 and 1 (as presented in the 3rd column of **Supplementary Data 9**). Among the top five cSL pairs, we selected two new combinations between highly specific inhibitor compounds that are marked to be *the most synergistic* based on the predicted cSL interactions - AKT1 and PIK3CA, and BCL2L1 and IGF1R (**Supplementary Data 9**) (**Supplementary Figure 7A**). Among the 29 patient-derived melanoma cell lines available, we selected the cell lines for each pair in which both the target genes are highly expressed, which include 501Mel commercial melanoma cell line and 3 PDCs for BCL2L1-IGF1R and 4 PDCs for AKT1-PIK3CA (**Supplementary Figure 7A**), where two of which overlap (**Supplementary Data 10**). We selected highly specific inhibitor compounds that target these genes, namely IGF1R inhibitor OSI906, Bcl-2 inhibitor ABT-263, AKT inhibitor MK-2206, and PI3K inhibitor GDC-0941.

Our experiment was performed in two-steps. First, we tested the synergism by varying the dose of one drug (8 doses) while keeping the concentration of the paired drug constant (5uM). This initial experiment confirmed that the drug pairs are synergistic in all cases tested (**Supplementary Figure 7B,C**, **Supplementary Data 11**).

Second, to systematically test the synergism, each drug pair was examined across 8 dose conditions with 1:1 dose ratio and the synergy values were calculated according to the Chou-Talalay method[32], using the CompuSyn software (ComboSyn, Inc., Paramus, NJ). The drug interaction is quantified by Combination Index (CI) values across different fraction of cells affected (Fa), where CI < 1, CI = 1, and CI > 1 indicate synergistic, additive, and antagonistic effects, respectively (see **Supplementary Figure 7D** for Fa-CI plots, and **Supplementary Data 12** for raw data).

Predicting *erlotinib* response in lung cancer patients

To compare the predictive performance of the a 76-gene epithelial-mesenchymal-transition (EMT) signature reported in the original study[33], we first identified top 76 significant SL-partners of EGFR based on their cSL-pair-score (Methods). We applied the resulting drug-cSL network to predict the response of EGFR wild-type 25 patients with recurrent or metastatic *Non-Small Cell Lung Cancer (NSCLC)* to the EGFR-inhibitor *erlotinib*[33,34]. The *erlotinib* cSL-score predicts the specific response to *erlotinib*: for an independent arm of the same trial, in which 37 NSCLC patients were treated with *sorafenib*, a VEGFR inhibitor, the predictions were not associated with months-to-progression in this case (log rank P>0.95, **Supplementary Figure 9C**). We observed that the expression levels of EGFR itself do not have a predictive signal (R = 0.03, P = 0.5), and even though mutated KRAS is a biomarker of resistance to EGFR-inhibitors[33], KRAS mutations are not significantly associated with progression-free survival (t-test P = 0.3), either. The performance of the drug-cSLi is on par with other supervised clinical drug response predictors applied previously to this data, which have achieved R = 0.61 and 0.56, P = 1.1E-3 and 0.01, when applied to all patients or only to KRAS wild-type patients[35]. Finally, DAISY or ncSL networks failed to predict the clinical response to *erlotinib* (log rank P=0.56 (ΔAUC=-=0.10) and log rank P=0.67 (ΔAUC=-0.01), respectively).

Threshold for gene underactivation

We selected the simplest possible approach that is free from assuming a specific background distribution to minimize the number of parameters needed. For every gene, across samples in each cancer type, we marked the bottom 1/3-quantile as under-expressed. We applied the exact same threshold (1/3-quantile) for determining inactivity from gene expression and SCNA data across all datasets analyzed. We verified that the outcome of our analysis does not depend on the threshold for gene under-activation using the METABRIC breast cancer cohort. We divided the patients into two groups of high cSL-score (top 50%) and low-cSL score (bottom 50%) and compared the difference in their survival using five different underexpression thresholds (**Supplementary Figure 10A)**, demonstrating that ISLE's predictive signal does not depend on the parameter choices for gene

underactivation. This analysis was performed using the core cSL network by applying FDR<0.1 to ISLE pipeline (**Figure 1B**).

Implementation of DAISY

DAISY[36] consists of three steps, (1) genomic survival of the fittest, (2) shRNA-based functional examination, and (3) pairwise gene co-expression. Of note, step (1) and (3) uses a set of similar data as ISLE's step I and step (2) uses a similar set of data as those used to construct ISLE's initial set II. To make a fair comparison between DAISY and ISLE, that is not biased with the types and size of the input dataset, we used the exactly same dataset for step (1) and step (2) of ISLE to implement DAISY. The SL partners were identified using FDR threshold of 0.2 in an analogous manner to ISLE following the DAISY's Methods described in the original paper[36]. DAISY SL-pair-score was calculated in an analogous manner as ISLE's cSL score, namely,

$$\text{DAISY SL pair score} = r_{(1)} + r_{(2)} + r_{(3)},$$

where *DAISY-SL-pair-score* is a qualitative measure combining the significance levels, $r_{(1)}$, $r_{(2)}$ and $r_{(3)}$ denote the rank-normalized values (between 0 and 1, with 1 representing a pair with the highest significance and 0 with the lowest) of the statistical significance levels across all gene pairs tested for step (1), (2), and (3) of DAISY, respectively.

Supplementary Note 2

We provide interactive maps of the network, which can be explored using the freely available Cytoscape software[37,38]. The maps visualize different subnetworks and include various gene properties and annotations, as well as alternative views that dissect the network hubs or genes with specific characteristics. The interactive maps are accessible through https://github.com/jooslee/ISLE/.

ISLE_clinical_SL_network_FDR_0.1.cys – an interactive visualization of the core cSLi obtained with FDR<0.1. The network includes genes (nodes) and their SLi (edges), and the largest subnetwork is displayed in **Figure 1B**.
ISLE_clinical_SL_network_FDR_0.2.cys – an interactive visualization of the full cSLi obtained with FDR<0.2. The network includes genes (nodes) and their SLi (edges), and the largest subnetwork is displayed in **Supplementary Figure 1**.
ISLE_drug_cSL_network.cys – an interactive visualization of the cSLi associated with drugs targets, namely, SLi between gene A and gene T, where gene T is a target of a drug. The network includes 232 SLi, involving 14 drug targets (blue), their corresponding 16 drugs (green), and their 207 SL-partners (red) used to predict treatment outcome of cancer patients (**Supplementary Figure 8**).

Supplementary Figures

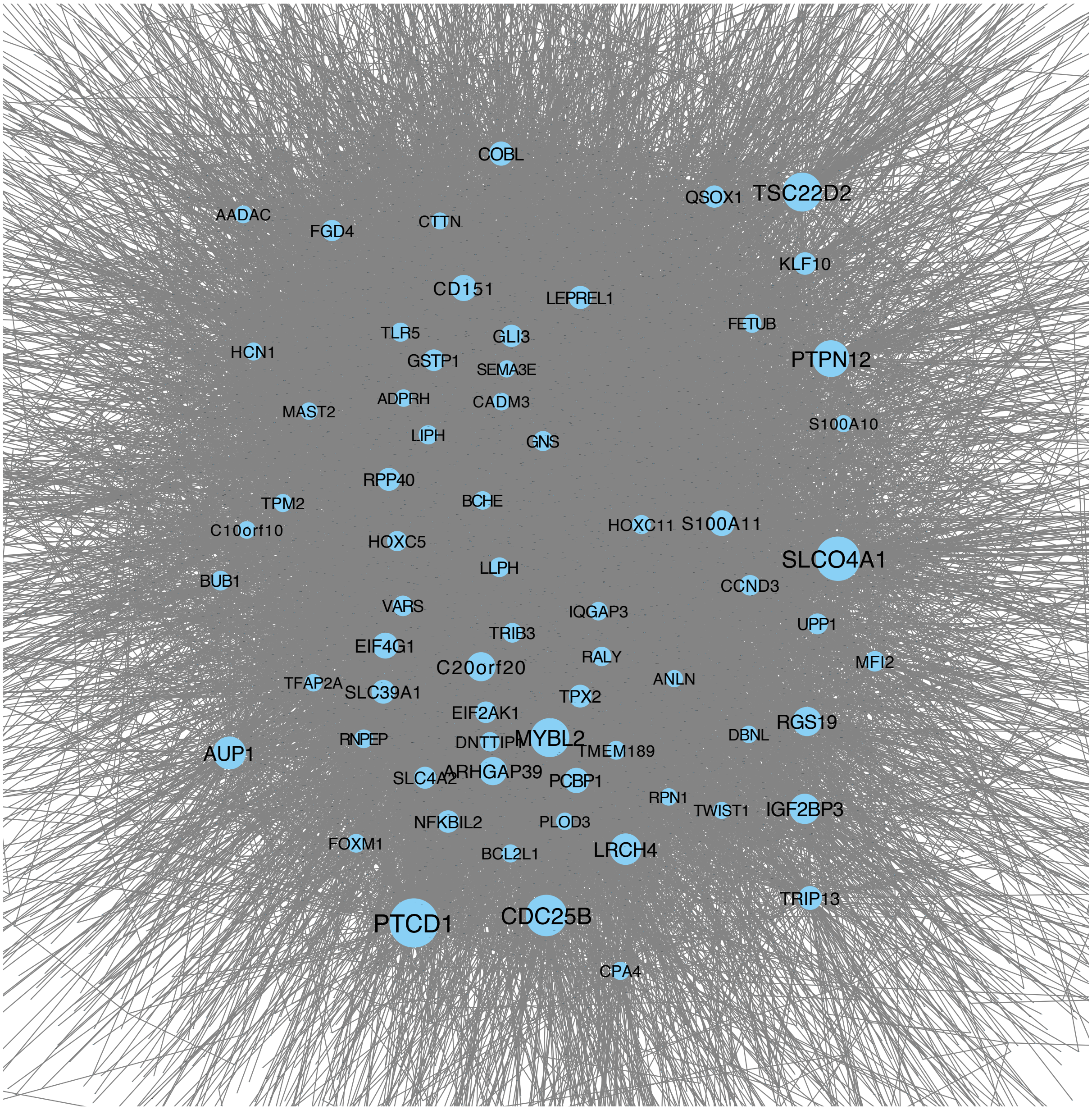

Supplementary Figure 1. Clinical SL network. The clinical SL network includes 21,534 interactions between 8,511 genes, where the gene names of degree >10 are marked; the size of nodes is proportional to the number of interactions they have. The interactive network is accessible at GitHub: https://github.com/jooslee/ISLE/ (ID: ISLE-reviewers, password: pass2ISLE).

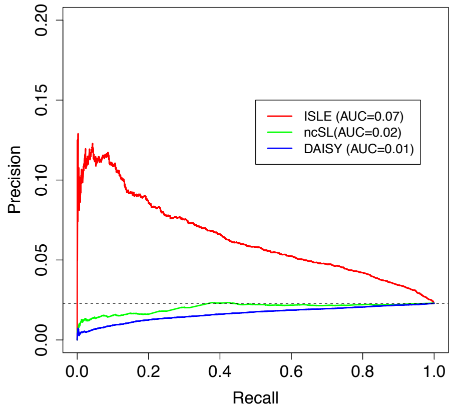

Supplementary Figure 2. Prediction of *in vitro* drug response using ISLE in comparison to DAISY in CCLE[39] collections. The precision-recall curve compares the prediction performance of ISLE (red) to that of ncSL (green) and DAISY (blue).

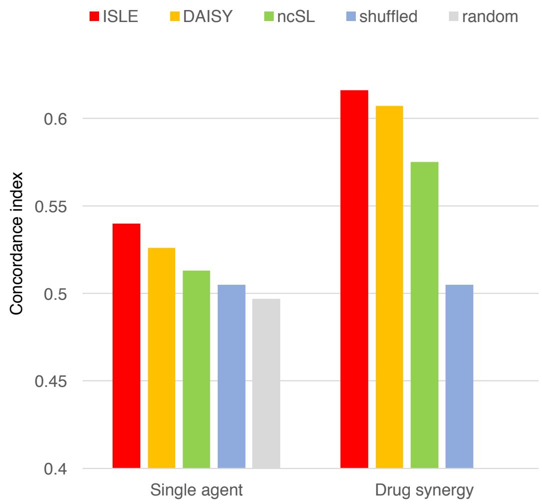

Supplementary Figure 3. Benchmarking ISLE using DREAM7 challenge data versus control SL networks. The figure shows the performance of ISLE (red) compared to DAISY(yellow), ncSL (green), and random SL pairs (blue, gray), where (i) SL-partners were randomly assigned (gray) and (ii) SL-partners of other drugs were assigned (blue) in predicting DREAM7 challenge drug response to single agents (left columns) and combinations (right columns). For drug combinations, only the randomly shuffled SL network of the drug targets were used as random control (blue). The performance was measured using weighted probabilistic concordance index for single agents and probabilistic concordance index for double treatments as was done in the original papers[27,28].

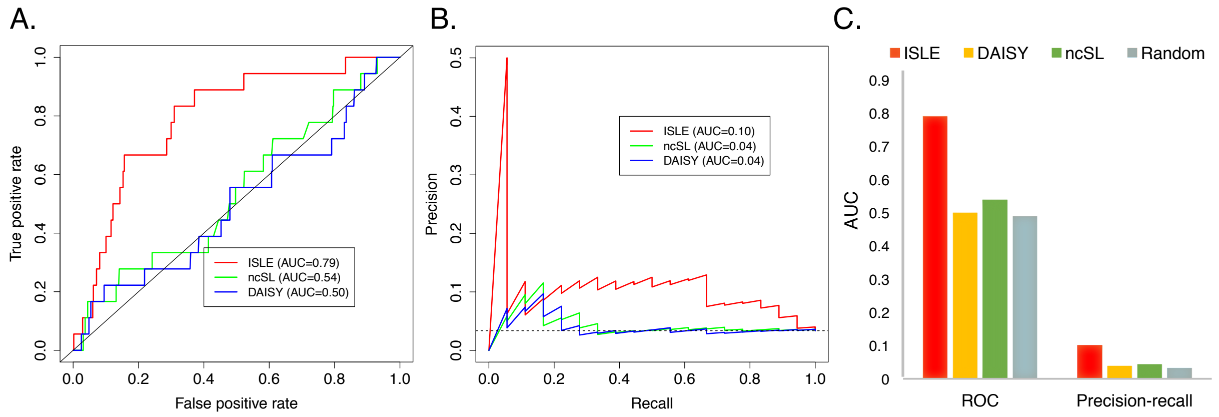

Supplementary Figure 4. Validating ISLE with *in vitro* drug synergy data. (A) ROC curves displaying the cSLi-based prediction accuracy of synergistic drug combination screens of a recent AstraZeneca-Sanger DREAM challenge (AUC=0.79). (B) Our predictor based on the significance of ISLE screenings (cSL-pair-score) shows significantly better precision-recall (AUPRC=0.10) compared to random predictor (AUPRC=0.033). (C) The AUC values of ROC and precision-recall curve are significantly higher for ISLE (red) compared to DAISY (yellow) and ncSL (green) as shown in (A-B), and this is not expected by the randomly shuffled SL networks (blue; empirical P<1E-3).

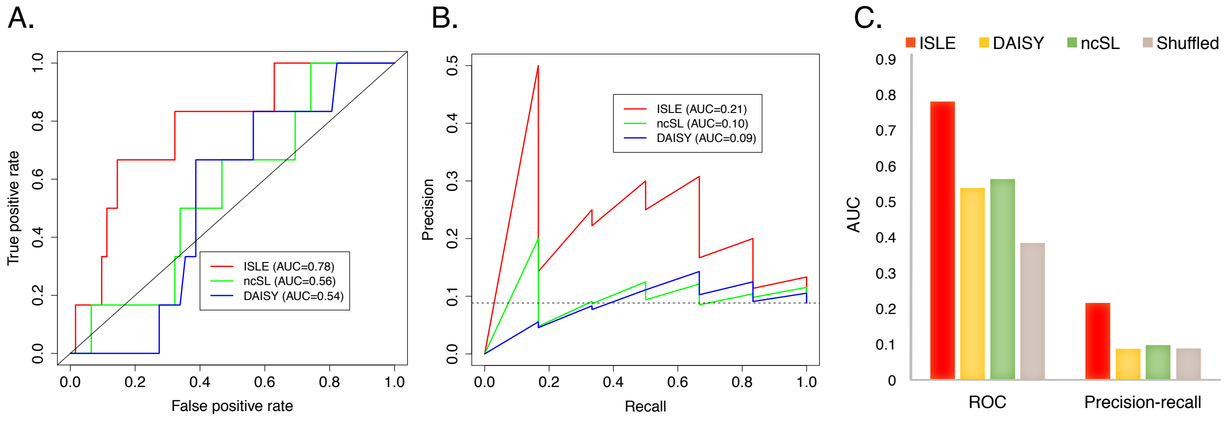

Supplementary Figure 5. Validating ISLE with *in vivo* drug synergy data. (A) The cSL predicted synergism of *in vivo* screening of synergistic drug combinations of 50 candidate gene pairs[26] using ISLE (red), DAISY (blue) and ncSL partners (that has support only from in vitro data; green). (B) Our predictor based on the significance of ISLE screenings (AUPRC=0.21) has significantly better precision-recall compared to random predictor (AUPRC=0.05). (C) The AUC values of ROC and precision-recall curve are significantly higher for ISLE (red) compared to DAISY (yellow) and ncSL (green) as shown in (A,B), and this is not expected by the randomly shuffled SL networks (gray; empirical P<1E-3).

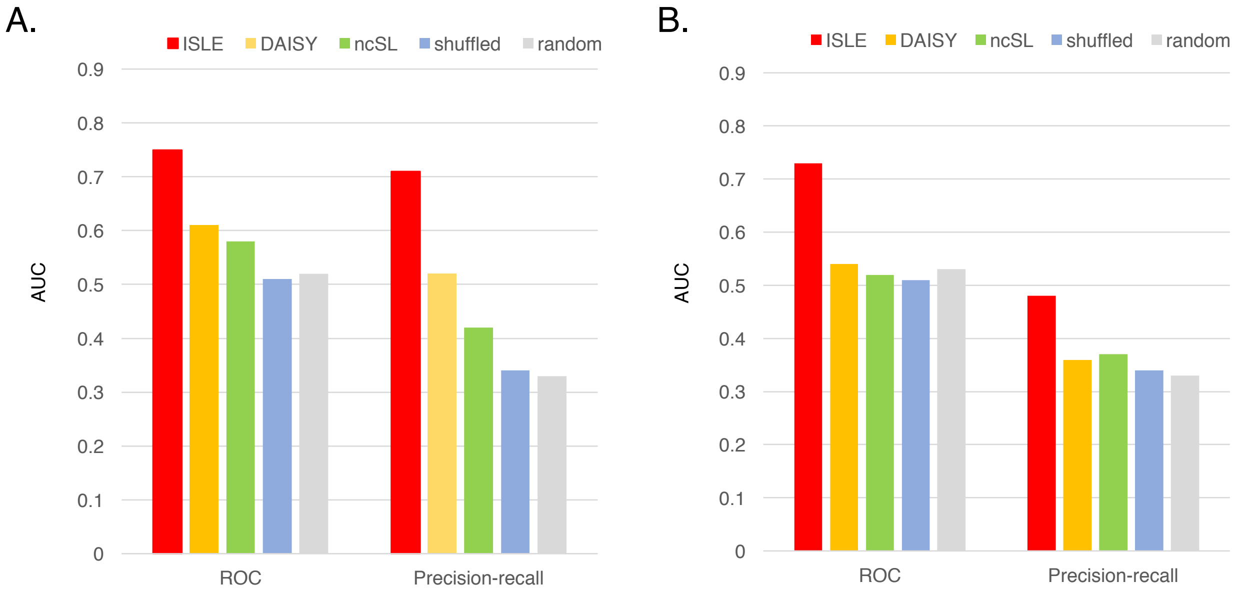

Supplementary Figure 6. ISLE's performance in large-scale drug prioritization prediction compared to control networks. Our prediction based on ISLE (red bars) shows higher prediction accuracy (quantified by the AUC of ROC curve (left) and Precision-Recall curve (right)) compared to the DAISY-SL (oange), ncSL (green), and random SL-networks (blue, gray), where (i) SL-partners are randomly assigned (gray) and (ii) SL-partners of other drugs were assigned (blue), with (A) in liv7k cell lines and (B) in BC cell lines (empirical P<1E-3).

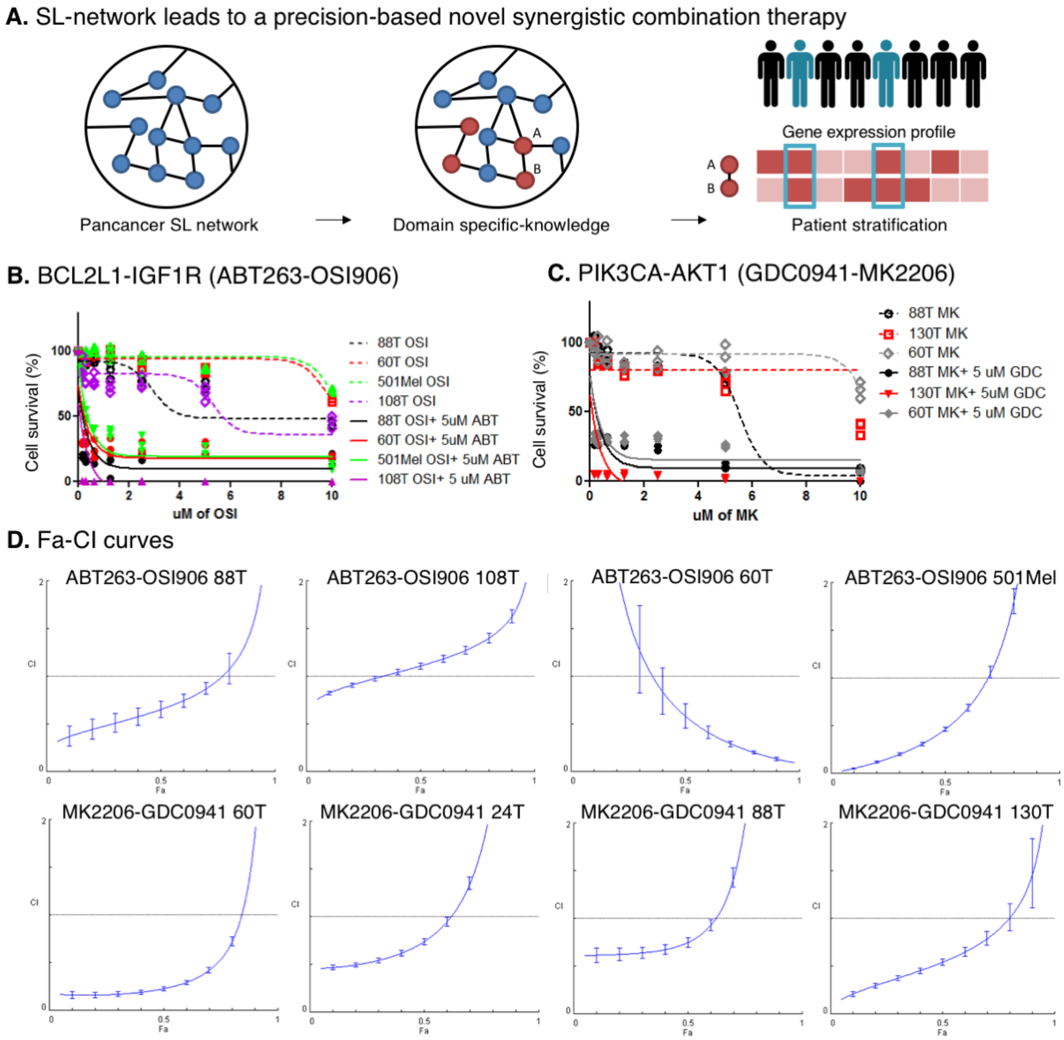

Supplementary Figure 7. SL-network predicts synergistic combinations in patient-derived melanoma cell-lines. A. The diagram summarizes how cSL-network can be used to develop a new precision-based combination therapy in specific cancer types. This can be done in two-step process: (1) From the pancancer drug cSL-network, the top significant cSL interactions between cancer type-specific single drug targets are selected based on the literature or experimental screens, and (2) based on the gene expression profile of patient tumor samples, the right patients are selected whose tumor shows expression of both target genes. **B,C.** The figures depict the dose-response curves of the predicted synergistic drug combinations of **(B)** ABT263 (BCL2L1 inhibitor) and OSI906 (IGF1R inhibitor) tested and **(C)** GDC0941 (PIK3CA inhibitor) and MK2206 (AKT1 inhibitor) from the first set of experiments, where the percentage of cell line survival (Y-axis) was measured at a varying doses of OSI906 (respectively MK2206), with and without ABT263 (respectively GDC0941) treatment at 5uM (X-axis). The dashed lines denote the percentage of cell line survival at varying levels of OSI906 (MK2206) without ABT263 (GDC0941) treatments, and the solid lines denote the percentage of cell line survival at varying levels of OSI906 (MK2206) in the presence of 5uM of ABT263 (GDC0941), and the different color denotes different cell lines including 501Mel and the patient-derived cell lines (88T, 108T, 60T, 24T, and 130T). The combined drug treatments are significantly more effective than the single treatments based on the analysis of variance in all cases ($P<1.0E-6$). **(D) Fa-CI curves of drug combination experiment in melanoma patient-derived cell lines.** The figures show Fa-CI curves of ABT263-OSI906 and MK2206-GDC0941 in 4 cell lines each (as denoted on the top of each Fa-CI curve). In 7 out of 8 cases, CI is below 1 for considerable ranges of Fa (e.g., IC<1 at Fa 50%).

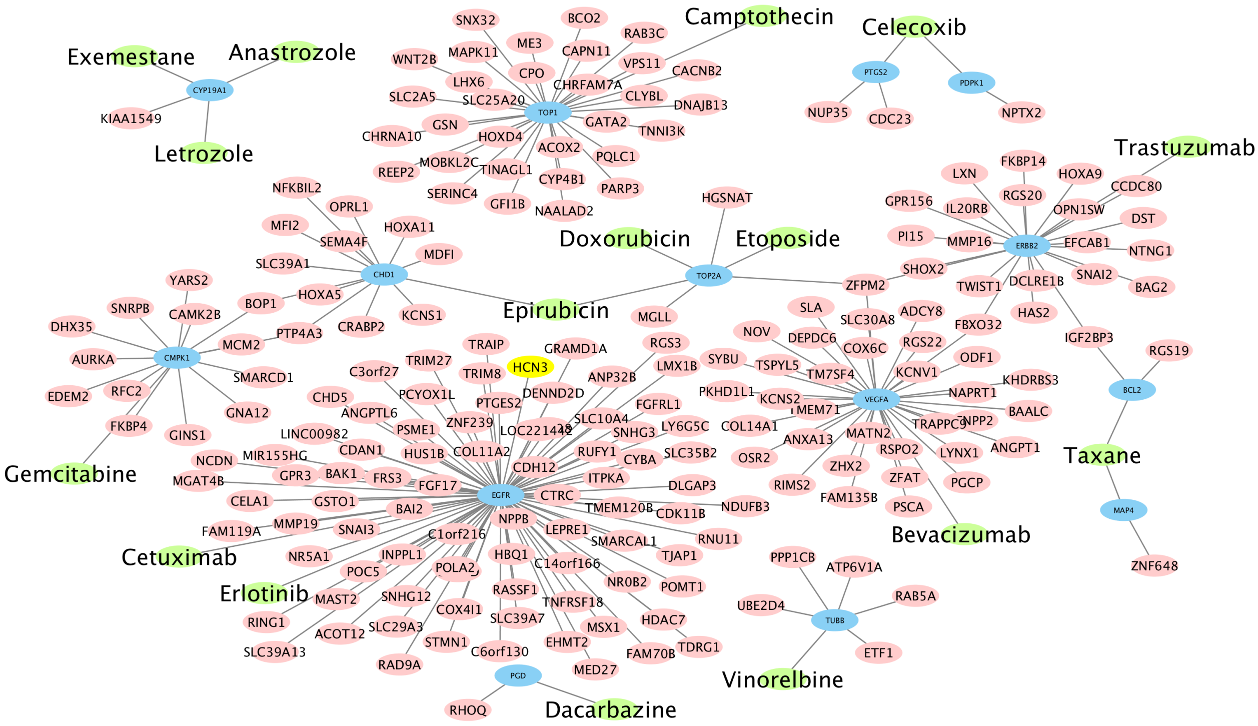

Supplementary Figure 8. Drug cSL network The figure shows drug cSL network that was used for patient drug response analysis (Sec 4), covering 232 cSL interactions between 14 target genes (blue) of 16 drugs (green) with 207 genes (red). The interactive form is accessible through https://github.com/jooslee/ISLE/.

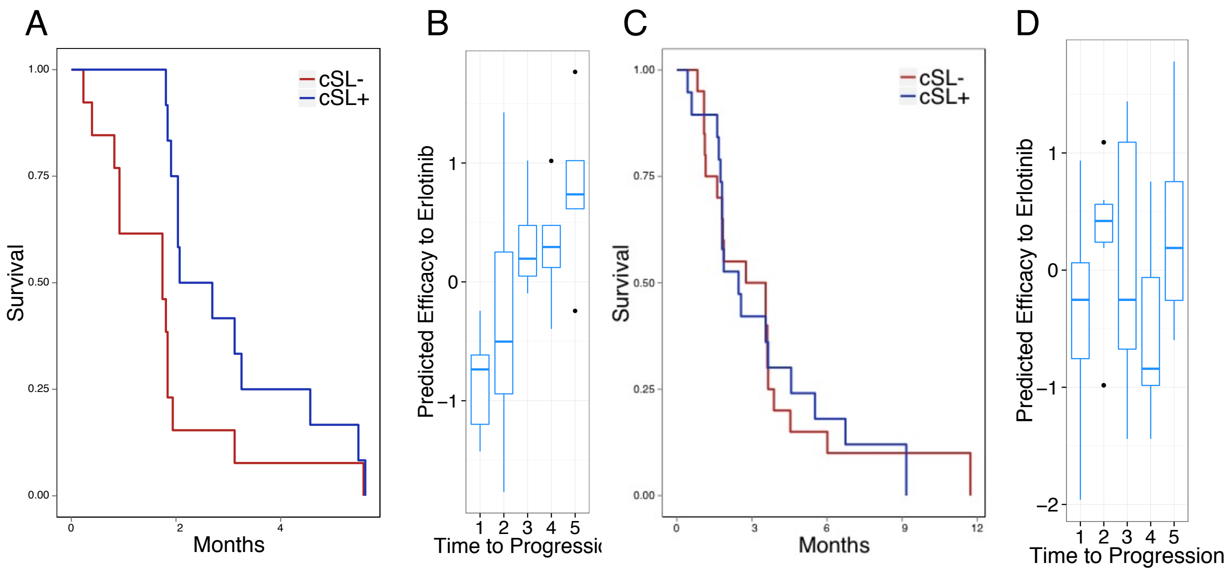

Supplementary Figure 9. Predicting patients' drug response to EGFR-inhibitor, *erlotinib*. (**A**) The KM plot shows that patients predicted as responders (blue, high-cSL-score: top 50-percentile) have a better prognosis than patients predicted as non-responders (red, low-cSL-score: bottom 50-percentile). This finding remains significant when the confounding factors including age and KRAS mutation are controlled (Cox hazard ratio =2.15 (P<6.5E-3), log rank P<0.03 (ΔAUC=0.23), empirical P<0.05). **(B**) We divided the 25 patients into 5 groups based on the time to progression, and find that it takes more time to progress in the patients whose normalized cSL-score (predicted efficacy) is high. (**C-D**) The control analysis for (A-B) using 37 *sorafenib* treated patients. The difference in survival based on cSL-score of Erlotinib is not significant (log rank P>0.95 (ΔAUC=-0.003)), and the time to progression does not show an association with the cSL-score.

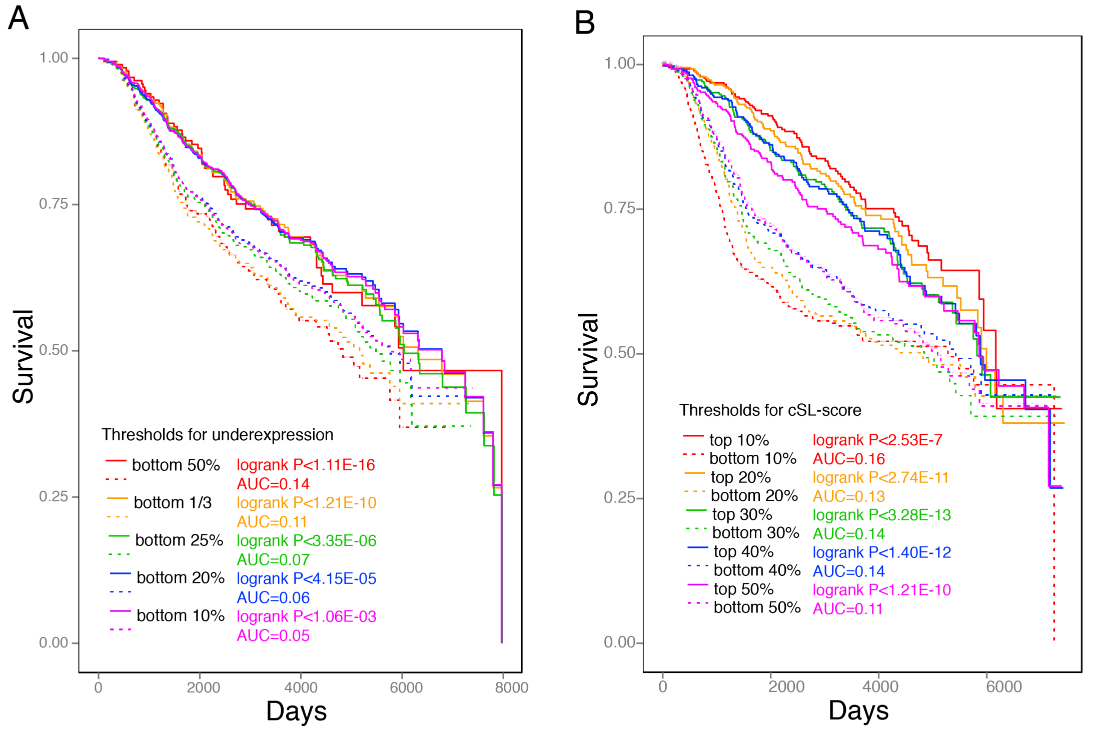

Supplementary Figure 10. Robust predictive signal of ISLE across different thresholds defining gene inactivation and high/low cSL-scores. **(A) Thresholds for gene underactivation:** The KM plots shows that patients with high cSL-scores (top 50%; solid line) have better prognosis than those with low cSL-scores (bottom 50%; dashed line) with the with five different gene under-expression thresholds (50% (red), 1/3 (orange), 25% (green), 20% (blue), and 10% (purple)). **(B) Thresholds for high vs low cSL-score groups:** The KM plots shows that patients with high cSL-scores (solid line) have better prognosis than those with low cSL-scores (dashed line), using five different cSL score thresholds, namely top/bottom 10% (red), 20% (orange), 30% (green), 40% (blue), and 50% (purple). The 1/3-quantile threshold was used to determine gene underexpression. Both analyses examined the core clinical-SL-network inferred with FDR<0.1 (**Figure 1B**).

Supplementary References

1 Whitehurst, A. W. *et al.* Synthetic lethal screen identification of chemosensitizer loci in cancer cells. *Nature* **446**, 815-819, doi:10.1038/nature05697 (2007).

2 Bommi-Reddy, A. *et al.* Kinase requirements in human cells: III. Altered kinase requirements in VHL-/- cancer cells detected in a pilot synthetic lethal screen. *Proc Natl Acad Sci U S A* **105**, 16484-16489, doi:10.1073/pnas.0806574105 (2008).

3 Lord, C. J., McDonald, S., Swift, S., Turner, N. C. & Ashworth, A. A high-throughput RNA interference screen for DNA repair determinants of PARP inhibitor sensitivity. *DNA Repair* **7**, 2010-2019, doi:http://dx.doi.org/10.1016/j.dnarep.2008.08.014 (2008).

4 Turner, N. C. *et al.* A synthetic lethal siRNA screen identifying genes mediating sensitivity to a PARP inhibitor. *EMBO J* **27**, 1368-1377, doi:http://www.nature.com/emboj/journal/v27/n9/suppinfo/emboj200861a_S1.html (2008).

5 Martin, S. A. *et al.* Methotrexate induces oxidative DNA damage and is selectively lethal to tumour cells with defects in the DNA mismatch repair gene MSH2. *EMBO Molecular Medicine* **1**, 323-337, doi:10.1002/emmm.200900040 (2009).

6 Luo, J. *et al.* A Genome-wide RNAi Screen Identifies Multiple Synthetic Lethal Interactions with the Ras Oncogene. *Cell* **137**, 835-848 (2009).

7 Steckel, M. *et al.* Determination of synthetic lethal interactions in KRAS oncogene-dependent cancer cells reveals novel therapeutic targeting strategies. *Cell Res* **22**, 1227-1245, doi:10.1038/cr.2012.82 (2012).

8 Toyoshima, M. *et al.* Functional genomics identifies therapeutic targets for MYC-driven cancer. *Proc Natl Acad Sci U S A* **109**, 9545-9550, doi:10.1073/pnas.1121119109 (2012).

9 Vizeacoumar, F. J. *et al.* A negative genetic interaction map in isogenic cancer cell lines reveals cancer cell vulnerabilities. *Mol Syst Biol* **9**, 696, doi:10.1038/msb.2013.54 (2013).

10 Wang, X., Fu, A. Q., McNerney, M. E. & White, K. P. Widespread genetic epistasis among cancer genes. *Nat Commun* **5**, 4828, doi:10.1038/ncomms5828 (2014).

11 Blomen, V. A. *et al.* Gene essentiality and synthetic lethality in haploid human cells. *Science* **350**, 1092-1096, doi:10.1126/science.aac7557 (2015).

12 Shen, J. P. *et al.* Chemogenetic profiling identifies RAD17 as synthetically lethal with checkpoint kinase inhibition. *Oncotarget* **6**, 35755-35769, doi:10.18632/oncotarget.5928 (2015).

13 Pathak, H. B. *et al.* A Synthetic Lethality Screen Using a Focused siRNA Library to Identify Sensitizers to Dasatinib Therapy for the Treatment of Epithelial Ovarian Cancer. *PLoS One* **10**, e0144126, doi:10.1371/journal.pone.0144126 (2015).

14 Srivas, R. *et al.* A Network of Conserved Synthetic Lethal Interactions for Exploration of Precision Cancer Therapy. *Mol Cell* **63**, 514-525, doi:10.1016/j.molcel.2016.06.022 (2016).

15 Wang, T. *et al.* Gene Essentiality Profiling Reveals Gene Networks and Synthetic Lethal Interactions with Oncogenic Ras. *Cell* **168**, 890-903 e815, doi:10.1016/j.cell.2017.01.013 (2017).

16 Han, K. *et al.* Synergistic drug combinations for cancer identified in a CRISPR screen for pairwise genetic interactions. *Nat Biotechnol*, doi:10.1038/nbt.3834 (2017).

17 Shen, J. P. *et al.* combinatorial CRISPR-Cas9 screens for de novo mapping of genetic interactions. *Nat Methods* (2017).

18 Cowley, G. S. *et al.* Parallel genome-scale loss of function screens in 216 cancer cell lines for the identification of context-specific genetic dependencies. *Sci Data* **1**, 140035, doi:10.1038/sdata.2014.35 (2014).

19 Cheung, H. W. *et al.* Systematic investigation of genetic vulnerabilities across cancer cell lines reveals lineage-specific dependencies in ovarian cancer. *Proc Natl Acad Sci U S A* **108**, 12372-12377, doi:10.1073/pnas.1109363108 (2011).

20 Marcotte, R. *et al.* Functional Genomic Landscape of Human Breast Cancer Drivers, Vulnerabilities, and Resistance. *Cell* **164**, 293-309, doi:10.1016/j.cell.2015.11.062 (2016).

21 Marcotte, R. *et al.* Essential gene profiles in breast, pancreatic, and ovarian cancer cells. *Cancer Discov* **2**, 172-189, doi:10.1158/2159-8290.CD-11-0224 (2012).

22 Aguirre, A. J. *et al.* Genomic Copy Number Dictates a Gene-Independent Cell Response to CRISPR/Cas9 Targeting. *Cancer Discov* **6**, 914-929, doi:10.1158/2159-8290.CD-16-0154 (2016).

23 Benjamini, Y. & Hochberg, Y. Controlling the False Discovery Rate: A Practical and Powerful Approach to Multiple Testing. *Journal of the Royal Statistical Society Series B-Methodological* **57**, 289-300 (1995).

24 Kim, H. & Park, H. Sparse non-negative matrix factorizations via alternating non-negativity-constrained least squares for microarray data analysis. *Bioinformatics* **23**, 1495-1502, doi:10.1093/bioinformatics/btm134 (2007).

25 Curtis, C. *et al.* The genomic and transcriptomic architecture of 2,000 breast tumours reveals novel subgroups. *Nature* **486**, 346-352, doi:http://www.nature.com/nature/journal/v486/n7403/abs/nature10983.html#supplementary-information (2012).

26 Gao, H. *et al.* High-throughput screening using patient-derived tumor xenografts to predict clinical trial drug response. *Nat Med* **21**, 1318-1325, doi:10.1038/nm.3954 (2015).

27 Costello, J. C. *et al.* A community effort to assess and improve drug sensitivity prediction algorithms. *Nat Biotechnol* **32**, 1202-1212, doi:10.1038/nbt.2877 (2014).

28 Bansal, M. *et al.* A community computational challenge to predict the activity of pairs of compounds. *Nat Biotechnol* **32**, 1213-1222, doi:10.1038/nbt.3052 (2014).

29 Gonzalez-Perez, A. *et al.* IntOGen-mutations identifies cancer drivers across tumor types. *Nat Methods* **10**, 1081-1082, doi:10.1038/nmeth.2642 (2013).

30 Forbes, S. A. *et al.* COSMIC: somatic cancer genetics at high-resolution. *Nucleic Acids Res* **45**, D777-D783, doi:10.1093/nar/gkw1121 (2017).

31 Cancer Genome Atlas, N. Genomic Classification of Cutaneous Melanoma. *Cell* **161**, 1681-1696, doi:10.1016/j.cell.2015.05.044 (2015).

32 Chou, T. C. Drug combination studies and their synergy quantification using the Chou-Talalay method. *Cancer Res* **70**, 440-446, doi:10.1158/0008-5472.can-09-1947 (2010).

33 Byers, L. A. *et al.* An epithelial-mesenchymal transition gene signature predicts resistance to EGFR and PI3K inhibitors and identifies Axl as a therapeutic target for overcoming EGFR inhibitor resistance. *Clinical cancer research : an official journal of the American Association for Cancer Research* **19**, 279-290, doi:10.1158/1078-0432.ccr-12-1558 (2013).

34 Kim, E. S. *et al.* The BATTLE Trial: Personalizing Therapy for Lung Cancer. *Cancer Discovery*, doi:10.1158/2159-8274.cd-10-0010 (2011).

35 Geeleher, P., Cox, N. J. & Huang, R. S. Clinical drug response can be predicted using baseline gene expression levels and in vitro drug sensitivity in cell lines. *Genome Biol* **15**, R47, doi:10.1186/gb-2014-15-3-r47 (2014).

36 Jerby-Arnon, L. *et al.* Predicting cancer-specific vulnerability via data-driven detection of synthetic lethality. *Cell* **158**, 1199-1209, doi:10.1016/j.cell.2014.07.027 (2014).

37 Cline, M. S. *et al.* Integration of biological networks and gene expression data using Cytoscape. *Nat. Protocols* **2**, 2366-2382, doi:http://www.nature.com/nprot/journal/v2/n10/suppinfo/nprot.2007.324_S1.html (2007).

38 Shannon, P. *et al.* Cytoscape: a software environment for integrated models of biomolecular interaction networks. *Genome Res* **13**, 2498 - 2504 (2003).

39 Barretina, J. *et al.* The Cancer Cell Line Encyclopedia enables predictive modelling of anticancer drug sensitivity. *Nature* **483**, 603-607, doi:10.1038/nature11003 (2012).
