# Supplementary material for: Harnessing synthetic lethality to predict the response to cancer treatment
Source: Nat Commun. 2018 Jun 29;9:2546. doi: 10.1038/s41467-018-04647-1 (PMC6026173; doi:10.1038/s41467-018-04647-1)
Supplement: Supplementary file 3 — Description of Additional Supplementary Files [file 41467_2018_4647_MOESM3_ESM.pdf]

Description of Additional Supplementary Files:

Supplementary Data 1. Experimentally identified gold standard SL interactions

Supplementary Data 2. Significant overlap between experimentally identified and inferred SL interactions

Supplementary Data 3. Fraction of initial pool of candidate SL pairs that pass each step of ISLE

Supplementary Data 4. Pathway enrichment of clinical SL-network

Supplementary Data 5. Fraction of experimentally identified and inferred SL sets that are supported by tumor data

Supplementary Data 6. Gene essentiality screen in liv7k cell line (Shanks essentiality in oral cancer data, % growth inhibition)

Supplementary Data 7. Drug response screen in liv7k cell line (Shanks drug response in oral cancer data)

Supplementary Data 8. Drug response screen in 7 breast cancer cell lines (Shanks drug response in breast cancer data)

Supplementary Data 9. Drug-cSL interactions between the genes involved in key pathways in melanoma

Supplementary Data 10. Expression level of the target genes in selected patient-derived cell lines

Supplementary Data 11. Experimental measurements of drug synergism with varying dose of one drug while keeping the dose of the paired drug fixed (Yardena data I)

Supplementary Data 12. Experimental measurements of drug synergism with varying dose of both drugs with 1:1 ratio (Yardena data II)

Supplementary Data 13. Drug to target gene mapping for patient drug response analysis

Supplementary Data 14. Summary of datasets used and their characteristics
